# Waiting for a digital therapist: three challenges on the path to psychotherapy delivered by artificial intelligence

**DOI:** 10.3389/fpsyt.2023.1190084

**Published:** 2023-06-01

**Authors:** J. P. Grodniewicz, Mateusz Hohol

**Affiliations:** Copernicus Center for Interdisciplinary Studies, Jagiellonian University, Kraków, Poland

**Keywords:** artificial intelligence, mental health chatbots, psychotherapy, therapeutic alliance, narrow vs. general AI, large language models, Cognitive Behavioral Therapy (CBT), conversational agents

## Abstract

Growing demand for broadly accessible mental health care, together with the rapid development of new technologies, trigger discussions about the feasibility of psychotherapeutic interventions based on interactions with Conversational Artificial Intelligence (CAI). Many authors argue that while currently available CAI can be a useful supplement for human-delivered psychotherapy, it is not yet capable of delivering fully fledged psychotherapy on its own. The goal of this paper is to investigate what are the most important obstacles on our way to developing CAI systems capable of delivering psychotherapy in the future. To this end, we formulate and discuss three challenges central to this quest. Firstly, we might not be able to develop effective AI-based psychotherapy unless we deepen our understanding of what makes human-delivered psychotherapy effective. Secondly, assuming that it requires building a therapeutic relationship, it is not clear whether psychotherapy can be delivered by non-human agents. Thirdly, conducting psychotherapy might be a problem too complicated for narrow AI, i.e., AI proficient in dealing with only relatively simple and well-delineated tasks. If this is the case, we should not expect CAI to be capable of delivering fully-fledged psychotherapy until the so-called “general” or “human-like” AI is developed. While we believe that all these challenges can ultimately be overcome, we think that being mindful of them is crucial to ensure well-balanced and steady progress on our path to AI-based psychotherapy.

## 1. Introduction

The mental health crisis is arguably among the most important global challenges that we are currently facing (1–3). Responding to it would require developing large-scale, high-quality, accessible, and affordable mental health care solutions (4). We might not be able to do this without benefitting from cutting-edge technology, including Artificial Intelligence (AI).

In recent years, AI has begun to be implemented in multiple domains of mental health care (5–7). AI-based solutions are used to improve the diagnosis of depression (8–10) and schizophrenia (11–13) and predicting treatment outcomes (14–17). Intelligent robots work with children with autism spectrum disorders (18) and elderly people suffering from dementia (19). Virtual reality avatars help patients confront their auditory hallucinations (20, 21). The list goes on.

One subdomain of mental health care where implementation of AI technology is both particularly challenging and promising is psychotherapy or “talk therapy” (22–31). It is difficult to find an uncontroversial definition of talk therapy acceptable to representatives of all therapeutic traditions. Nevertheless, as a first approximation, we can appeal to the following characterization offered by American Psychological Association (APA) (32), according to which psychotherapy is:

communication between patients and therapists that is intended to help people: (i) find relief from emotional distress, as in becoming less anxious, fearful or depressed, (ii) seek solutions to problems in their lives, such as dealing with disappointment, grief, family issues, and job or career dissatisfaction, and (iii) modify ways of thinking and acting that are preventing them from working productively and enjoying personal relationships.^1^

The APA characterization goes on to differentiate therapy from “talking with a friend.” While both a therapist and a friend may be willing to listen about our problems, only therapists are “trained professionals with specialized education and experience in understanding psychological problems” (32). Moreover, in contrast with friendship, therapy is non-symmetrical, and focuses solely on the client’s well-being. Finally, therapy takes place in a structured setting, typically there is an agreement between the client and therapist regarding regular meeting times, the length of each meeting, etc.

Given the rapid development of ever more impressive AI technologies, it is only natural to wonder whether AI already is, or will be in the future, able to conduct psychotherapy, and thus challenge the traditional conceptualization of psychotherapy as the relationship between two flesh-and-blood persons in which one cures the other. The closest we get to AI-based psychotherapy these days are the interventions delivered by mental health chatbots, many of which are based on relatively simple dialogue systems (34). In the next section (Section 2), we briefly discuss why several authors suggest that – even though they are sometimes oversold as offering fully fledged psychotherapy – currently available chatbots are falling short of achieving this goal. But what about chatbots available in 2, 5, or 10 years? As proved by the impressive performance of ChatGPT produced by OpenAI-Microsoft, interactions with CAI based on so-called “Large Language Models” (LLMs) start becoming deceptively similar to conversations with another human being. Moreover, while some authors are cautious about the abilities of CAI based on LLMs (35), others go as far as to claim that appropriately prompted CAIs are able to perform complex reasoning (36) or even manifest abilities similar to what psychologists call Theory of Mind, i.e., the ability to assign mental states (such as beliefs, desires, and intentions) to other agents (37, however, see 38). Does this mean that fully fledged psychotherapy delivered by CAI is around the corner? This is not so simple. On our way to developing CAI systems capable of delivering psychotherapy, we will encounter problems and obstacles that might be impossible to overcome by simply increasing the computational power of AI algorithms or training data sets of LLMs. The goal of this paper is to characterize three of them.

The first (discussed in Section 3) is *The Problem of a Confused Therapist*. The gist of the problem is that different therapeutic traditions conceptualize the process of psychotherapy differently, often disagreeing about what a therapist should do while conducting therapy. Moreover, there is heated discussion regarding the effectiveness of different therapeutic approaches, and it is unclear whether we will be able to develop an artificial system delivering the processes, techniques, and interventions that make psychotherapy effective unless we first better understand which processes, techniques, and interventions make psychotherapy effective. Clinical psychologists have tried to answer this question for the last 100 years, and we still seem to be far away from a fully satisfactory solution. Part of the problem is that it is not obvious to what extent the active ingredients in effective psychotherapy are specific techniques and interventions as opposed to such common factors as, e.g., a supportive therapeutic relationship.

This leads to the second problem – *The Problem of a Non-human Therapist* (Section 4). It has been traditionally assumed that psychotherapeutic care takes place within the frame of a relationship between two (or more) people. Can the “human aspect” of psychotherapy be reconstructed in the relationship between a person and an artificial system, e.g., by breaking it down into constituents such as trust, empathy, respect, mutual understanding, etc., which could later be emulated by an AI?

The third problem (Section 5) is *The Problem of a Narrowly Intelligent Therapist*. Despite the hype and enthusiasm around AI research, at least in the proximate future, we are stuck with narrow AI technologies. Narrow (as opposed to general) AI systems are very effective but only in performing well-delineated and clearly constrained tasks, such as image recognition, speech processing and generation, or even driving a car. The question is whether conducting psychotherapy is a task that can be so clearly delineated (or whether it can be divided into a set of such tasks). If not, it does not seem that we will be able to develop AI systems conducting fully fledged psychotherapy unless we first build a general AI, i.e., AI which – very much like a human – would be able to apply its intelligence to an unrestricted set of tasks.

## 2. Existing mental health chatbots

With more than 10,000 available options, the market of mental health apps is booming (39). Most of them implement relatively simple functionalities, such as mood, progress, and medication tracking, journaling, or easy access to prerecorded guided meditations and breathing exercises. However, in this section, we want to focus on the subset of most pioneering among these apps – mental health chatbots (6, 24, 26–31, 40).

Mental health chatbots are dialogue systems designed to interact with a human in real time in a way that mimics human–human conversation. To get a better grip on how mental health chatbots work, it will be useful to take a closer look at a fragment of an exemplary conversation with Woebot – one of the most popular and advanced chatbots designed to implement principles of Cognitive Behavioral Therapy (CBT) (41).^2^

1. Woebot: Can you say more about what’s going on?
2. User: I’m feeling bad.
3. Woebot: This sounds like a problem with feeling low, does that sound right to you?
4. User: [picking one of the available continuations] Yeah, that’s right.
5. Woebot: Sadness can make the smallest tasks seem like unclimbable mountains, but I want you to know: I’m here to help and we can get through this together.
6. User: [picking the only available continuation] Yeah.
7. Woebot: There’s two ways we can go about this. I can help you challenge your thinking, or we can focus on things you can do right now.
8. User: [picking one of the available continuations] Thought challenger.
9. Woebot: When you are feeling low, it can be helpful to understand how your thoughts affect your emotions. So we are going to take a close look at your thinking to help you feel better.
10. User: [picking the only available continuation] Okay.
11. Woebot: Before we dive in, a quick reminder: your mind is a powerful, incredible thing! It can solve problems, make plans, and process information at warp speed. But sometimes, our turbo-charged brains jump to conclusions that aren’t 100% true.
12. User: [picking one of the available continuations] Makes sense.
13. Woebot: Psychologists call these automatic negative thoughts – harsh ideas or statements that pop into your mind as you navigate daily life. Some people compare them to put-downs from a tiny character, like a villain or bully, that lives inside their head.
14. User: [picking the only available continuation] I see.
15. Woebot: Often these thoughts can be distorted, which means they make things feel worse or more extreme than they actually are. But once you learn to catch these thoughts and check them for distortions, you can change them so they are fairer, more balanced, and truer to reality.

Already in this short conversation, Woebot mimics a lot of elements of an actual therapy session. Just as a human therapist might do, it makes sure that it has understood the user correctly [3], normalizes the user’s experience (“Sadness can make the smallest tasks seem like unclimbable mountains), and suggests a form of the therapeutic alliance (“we can get through this together”) [5]. It also offers elements of psychoeducation by explaining that thoughts influence our emotions [9] and that our brains fall prey to cognitive errors [11], and by outlining the concept of “automatic negative thoughts” with the metaphor of “put-downs” from a “villain or bully” [13]. Finally, it introduces the basic idea behind one of the main therapeutic processes of CBT, i.e., cognitive restructuring (42), by suggesting that we should try to “catch,” “check,” and “change” our distorted thoughts [15]. In the further course of the conversation, Woebot guides the user through the process of restructuring, inviting them to recognize and revise distortions underlying a particular thought. For example, when a user inputs “Nobody loves me,” the exemplary conversation continues:

1. Woebot: Consider your thought “Nobody loves me.” Does it assume that because one bad thing happened, things are going to stay bad from here on out?
2. User: [picking one of the available continuations] Yes.
3. Woebot: Good catch. This distortion is called overgeneralization. If you make assumptions about your entire life based on a single event or situation, you are probably overgeneralizing.

Finally, Woebot invites the user to reformulate the thought in a way omitting these distortions, thus concluding a short intervention implementing the process of cognitive restructuring.

Several ethical problems involved in the use of mental health chatbots have been already raised (28–30, 43–46). They include, e.g., (i) concerns regarding data privacy, (ii) the risk of bypassing rather than fighting the stigma related to mental health issues by encouraging users to keep their struggle in the privacy of the interaction with their phone, and (iii) the lack of control over feedback and recommendations users receive from the app. These and other related risks are already discussed in considerable detail (28–30, 43–46) and we will not repeat these arguments here. Instead, we will focus on the question central to our current discussion: “what exactly do mental health chatbots do?”

In review articles devoted to therapeutic CAI we read that among the functions of mental health chatbots there is “delivering evidence-based psychological interventions” (26) [p. 1]; “providing cognitive behavioral therapy (CBT)” (31) [p. 459] and that “[t]he most common use of chatbots was delivery of therapy, training, and screening” (40) [p. 6]. Companies developing such chatbots are increasingly cautious not to characterize the services offered by chatbots as psychotherapy. For example, on its website, Woebot is presented as “your personal mental health ally,” not a therapist (41). However, the same website mentions that it helps “deliver individual support through interactive and easy-to-use therapeutic solutions” and that “There’s no such thing as appointments or waiting rooms here,” which clearly indicates a visit to one’s therapist as opposed to, e.g., using a self-help book, as the appropriate comparison class for Woebot. In a similar fashion, another popular product of this type – Wysa – is presented as “an AI chatbot that leverages evidence-based cognitive-behavioral techniques (CBT) to make you feel heard” (47). It is unclear whether “leveraging” therapeutic techniques equals “using” them and whether using therapeutic techniques is assumed to equal delivering psychotherapy. Among the benefits of using Tess – yet another mental health chatbot developed by X2 Foundation – we find “Effective: Most people report that they prefer Chat With Tess over traditional therapy…” and “Affordable: Support from Tess is 98% cheaper than face-to-face therapy” (48). Again, without explicitly calling Tess a “therapist,” X2 Foundation clearly suggests that it is appropriate to compare using their chatbot to attending human-delivered psychotherapy.

The lack of transparency regarding the actual nature of services provided by chatbots is probably most visible in what we may call “The Efficacy Overflow Argument,” often implicitly conveyed in the marketing of mental health chatbots (29). Here is the general form of the argument:

The efficacy overflow argument: (1) Chatbot C is based on the principles of a psychotherapeutic approach P. (2) There is evidence for efficacy of P. (3) Therefore, we should consider C to be evidence-based.

The argument is invalid. The fact that a given psychotherapeutic approach is effective when the therapy is conducted by a well-trained and experienced therapist over the course of multiple one-to-one sessions does not mean that the techniques or interventions based on the principles of this approach will be effective psychotherapy when administrated by a chatbot.

At the same time, a growing body of research shows that interactions with chatbots can contribute to improving their users’ mental health and quality of life, especially if the specific needs of the user match with chatbot capabilities (22, 24, 27, 31, 34, 40, 49–56). While these studies differ in terms of evaluation methods, in a recent meta-analysis of 32 randomized controlled trials, Yuhao He and colleagues (57) found that CAIs proved to be effective, in particular on a proximal time scale, in reducing depressive and anxiety (both general and specific) symptoms, preventing stress, general distress, and negative affect, and improving well-being. In light of these findings, the authors conclude that, “in the post-epidemic and digital eras, CAIs will likely play a significant role and contribute significantly to the new health transformation, in our care” [p. 15].

But can we say that the kind of psychological support offered by chatbots is already equivalent to psychotherapy, considered traditionally as a relationship between two persons or agents in which one of them heals or cures the other one (30, 45)? In a recent paper, Jana Sedlakova and Manuel Trachsel (28) argue that we should think about a conversational chatbot as “new artifacts” lying on the spectrum between therapeutic tools and therapists. According to these authors, chatbot is not a therapist because it is not a subject or agent. But it is not a mere tool either because it “might be experienced and treated as if it was a subject or agent” (28) [p. 4]. Unlike human therapists, existing chatbots cannot engage in normal human discursive practices, characterized by the ability of giving and asking for reasons, as well as understanding and explaining the concepts one uses. As such, according to Sedlakova and Trachsel, they cannot facilitate the acquisition of insight or self-understanding – one of the central elements of a psychotherapeutic process. On the other hand, as we argue in (58), interactions with a chatbot implementing CBT techniques might put a user in a better position to recognize “the connections between one’s emotions, motivations, thoughts, and behavior, past and present, including one’s interpretations of and relations with others” (59) (pp. 154–5), which is arguably at least an element of the process of acquiring self-understanding.

To sum up, we think that Sedlakova and Trachsel and other authors are right in raising the question of whether existing chatbots deliver support equivalent to traditional psychotherapy. We also agree with them that, in the foreseeable future, chatbots should be used cautiously; ideally as supplements for human delivered psychotherapeutic care [cf. (5, 26, 31)]. On the other hand, having in mind the growing effectiveness of existing mental health chatbots in reducing psychiatric symptoms and improving the well-being of their users as well as witnessing the current boom of CAI technologies along with their expected development over the next few years, it is worth considering what conditions would have to be met and what challenges overcome for us to be able to call an interaction with an artificial intelligence “psychotherapy.” This is the goal for the rest of this paper.

## 3. The problem of a confused therapist

The field of psychotherapeutic care is by no means monolithic. Prochaska and Norcross (60) estimate that there are now more than 500 different psychotherapeutic approaches. Most of these approaches belong to one of the main psychotherapeutic traditions, e.g., psychoanalytic/psychodynamic, existential, person-centered, behavioral, cognitive, etc. Principles underlying some of these traditions are more or less compatible with each other, which enabled the creation of therapeutic modalities benefitting from more than one of them. A prominent example here is Cognitive Behavioral Therapy (CBT) – undoubtedly one of the most popular modern therapeutic orientations (61). Principles underlying some other traditions (e.g., the psychoanalytic and the behavioral tradition) seem so far apart that it is much more difficult to think about their productive combination [which is not to say that such attempts have not been made (62)].

Different therapeutic traditions conceptualize human mental suffering differently. For example, while psychoanalytic tradition focuses on unresolved internal conflicts, cognitive tradition focuses on maladaptive beliefs, and patterns of thinking. Such theoretical differences result, in turn, in different repertoires of clinical processes and techniques – to put it simply, therapists working in different traditions do different things. For example, while we may expect a psychoanalyst to work with their client using free associations, dream interpretation, or analysis of transference, a CBT therapist will rather appeal to such tools as cognitive restructuring or exposure. In sum, specific therapeutic orientation influences the therapist’s way of “(a) generating hypotheses about a client’s experience and behavior, (b) formulating a rationale for specific treatment interventions, and (c) evaluating the ongoing therapeutic process” (63) [p. 412].

On top of that, the question that has loomed over the whole field for the last 100 years is whether different psychotherapeutic orientations (to the extent to which they are effective) are effective thanks to their specific features, or the so called “common” or “non-specific” factors. Since the publication of Rosenzweig (64), different authors suggested dozens of lists of common factors potentially responsible for making different therapies effective independently of what specific techniques they involve (65–70). For example, Weinberg (71) discusses such common factors as: (i) the therapeutic relationship between a therapist and a client/patient, (ii) expectations of therapeutic success, (iii) client’s confronting or facing the problem they struggle with, (iv) experience of mastery or control over the problematic issue, and (v) attribution of therapeutic success or failure to internal causes (e.g., changes in client’s coping skills) rather than external causes (e.g., therapist’s abilities and techniques).

This leads to the first problem on our way towards a fully fledged AI-based psychotherapy:

**The Problem of a Confused Therapist**: Can we develop artificial systems capable of conducting effective psychotherapy, given our limited understanding of the necessary components of a therapeutic process and factors that make psychotherapy effective?

This problem is relevant to the quest of achieving fully fledged AI-based psychotherapy but not specific to it. We face the same problem in the case of training of future therapists in general. Which orientation should they choose? Should they become Cognitive Behavioral, Gestalt, or psychodynamic therapists? Or should they pick yet something else from the plethora of options?

Research suggests that the decision to choose one’s therapeutic orientation is based, among others, on such factors as personality (72), individual learning styles (73), and value preferences (74). But in the case of developing artificial therapists, we will have to make this decision for them, either by hard-wiring them with principles of a given therapeutic modality or by training AI algorithms on data from sessions in which a particular therapeutic modality is implemented (75). Moreover, assuming that we would like to train our algorithms on data coming from successful psychotherapies, we will have to decide how we want to understand “success” in psychotherapy. Is it identical with symptom reduction? If yes, which ones and measured on what scale? Or maybe we should rather identify it with the improvement of a client’s functioning and general well-being (76)?

Furthermore, each therapist faces the question of whether they should be faithfully applying the methods of a therapeutic orientation they have been trained in or whether they should mix it with other methods, techniques, and processes that they find fit. According to Prochaska and Norcross (60), most therapists declare that they integrate methods from different therapeutic orientations. Should artificial therapists also be integrative or eclectic? Should we thus – on principle – train them on data from different therapeutic traditions? Additionally, each therapist must individually find their balance between specific therapeutic techniques and methods they apply during session, and less specific work such as nurturing clients’ sense of mastery and control. How should we go about achieving this balance in the case of artificial therapists? Prochaska and Norcross (60) point out that “Without a guiding theory or system of psychotherapy, clinicians would be vulnerable, directionless creatures bombarded with literally hundreds of impressions and pieces of information in a single session” [p. 4]. This is equally true about artificial therapists.

On the other hand, integrative approaches, “characterized by dissatisfaction with single-school approaches and a concomitant desire to look across school boundaries to see what can be learned from other ways of conducting psychotherapy” in order “to enhance the efficacy, efficiency, and applicability of psychotherapy” (77) [p. 4], may prove useful in our future attempts to develop new, and improve existing, therapeutic interventions delivered by CAIs. A notable example of one such attempt is the chatbot MYLO (78), which implements the core principles of a transdiagnostic, integrative therapy called Method of Levels (MOL) (79).^3^ MOL is based on an all-encompassing psychological theory: Perceptual Control Theory (80, 81), according to which the most important principle guiding life – from the level of basic biological functioning all the way up to mental health and well-being – is control. Psychological distress experienced by people seeking psychotherapeutic help results from the emergence of an internal conflict, which triggers a loss of control. According to the assumptions underlying MOL, all that clients need to be in a position to resolve such a conflict and regain control is for someone to “(1) help them to talk about the problem at length, in detail and in the present moment, thereby sustaining their attention to it, and (2) to notice disruptions in their speech and behaviour as they describe the problem, such that the client can shift their attention to aspects of the problem they may otherwise have missed” (82) [p. 140]. At least to some extent, this intervention has been implemented in the chatbot MYLO, which simply asks its users a series of questions, thereby creating a context in which a client can explore and resolve their internal conflict. Chatbots such as MYLO, if effective, can constitute proof of concept that solving The Problem of a Confused Therapist can proceed not by creating more sophisticated dialogue systems capable of deliver complicated therapeutic procedures but by relying on relatively simple therapeutic interventions. Even though the preliminary results are promising (78, 83, 84), more comprehensive studies on bigger and more diversified groups of users are in order.

Simultaneously, judging by the state of the field of mental health chatbots, the therapeutic orientation considered to be most promising for AI-based therapy is CBT. CBT, as delivered by a well-trained human specialist, is effective in the treatment of a broad set of diagnoses (85). It is also among the forms of therapy that bring positive results in a relatively short time. Moreover, the basic idea of CBT – that “self-relevant thoughts, evaluations, and beliefs are key contributors to the development and persistence of psychopathological states” (42) [p. 23] – is simple and elegant. At least to some extent, CBT can be broken down into a set of techniques that focus on identifying, challenging, and substituting such maladaptive thoughts and beliefs. This makes CBT relatively simple to operationalize (86). Thus, the assumption of many chatbot developers is that as long as chatbots help their users identify, challenge, and substitute their maladaptive cognitions – together with some additional skills and mindfulness training, and behavior activation – they *deliver* CBT (87).

But – many psychotherapists would suggest – even in the case of CBT, therapy is more than just techniques (88, 89). In fact, a whole chapter of one of the founding CBT textbooks (90) is devoted to the therapeutic relationship. Such a relationship is characterized by “warmth, accurate empathy, and genuineness” (90) [p. 45]. This leads us to the second problem on the way towards fully fledged AI-based psychotherapy.

## 4. The problem of a non-human therapist

As noted in Section 2, psychotherapy has traditionally been framed as a relationship between two persons or agents. These two characteristics are not on par. While we might be inclined to reserve the term “person” for human persons/people (91) it is less controversial to speak about “artificial agents,” assuming that “an agent is a being with the capacity to act, and ‘agency’ denotes the exercise or manifestation of this capacity” [(92), cf. (93)]. Existing mental health chatbots are obviously not persons, and probably not even agents, given how restricted and limited their capacity to act. But this might not be the case for the artificial therapists of the future. Therefore, the second challenge on the way towards AI-based psychotherapy is the following:

**The Problem of a Non-human Therapist**: Can a non-human agent conduct psychotherapy, given that, additionally (or primarily) to delivering specific techniques, it requires building a therapeutic relationship?

According to most authors, psychotherapy is much more than the delivery of specific techniques. It is first and foremost an interpersonal relationship (30, 45, 94, 95). This raises concerns regarding the possibility of fully fledged AI-based psychotherapy (26, 28–30, 45, 96).

However, instead of thinking about the lack of human–human relationship as an insurmountable obstacle, it might be better to think about it as a challenge. Ideally, everyone struggling with a mental health problem would have easy access to a well-trained human therapist, who will be additionally able to devote their clients as much time as necessary. Unfortunately, with a global median of 13 mental health workers per 100,000 population (62.2 in high-income countries) (97), and the estimate of 70% of people with mental illness receiving no treatment from health care staff (98), this scenario is unrealistic. Therefore, without neglecting the importance of human–human relationship, and without abandoning the efforts to increase the number of mental health workers *per capita*, we should actively seek alternative solutions.

It seems that there are three practical strategies to choose from, when confronted with The Problem of Non-human Therapist. Here, we will just put these strategies forward, leaving the task of their careful assessment for another occasion.

The first strategy – *Deflation* – involves deflating the role of a therapeutic relationship in the therapeutic process. Sure – a supporter of this strategy can say – when the psychotherapy involves two people, a deep relationship is likely to appear between them. In fact, this would be true about all prolonged interactions between two people – this is a part of our social set-up. The therapeutic relationship differs from other relationships, such as friendship, and a skilled therapist will steer it in such a way that it is most beneficent to achieving the therapeutic goals. But in the case of artificial therapists, such a relationship might be missing. We used to think about psychotherapy as involving a therapeutic relationship, because we used to think about it as involving humans. But the reality of psychotherapy of the future might be different, and we should not be stuck to our old standards, according to which therapeutic relationship is necessary for the psychotherapeutic process to occur. Maybe, instead of focusing on what is missing, we should focus on what other resources we have? It is true that the contribution of a therapeutic relationship to the overall efficacy of psychotherapy will be lost. But maybe we can regain it in other ways? For example, despite not being able to build a therapeutic relationship, artificial therapists may be much more skilled and consistent in delivering therapeutic techniques than humans, and thus at least as effective as human therapists.

One more thing worth keeping in mind in this context is that we already know some helpful psychological interventions that do not require a therapeutic relationship. A notable example is various writing techniques, which turn out – at least in certain populations – to be effective in reducing psychological distress (99). Therefore, e.g., Carey et al. (100), point out that while a therapeutic relationship might be a key component of effective psychological intervention (they go as far as to suggest that “it might be sufficient” for achieving the positive therapeutic effect; p. 48), it is not necessary. Even if it turns out that it is impossible to build a therapeutic relationship between a user and a CAI, we still might focus on training chatbots to use interventions that do not require such a relationship.

The second strategy is *Mimicry* [cf. (28)]. According to this strategy, what is important is not whether there *is* a therapeutic relationship, but whether the client *thinks* that there is such a relationship. In Section 2, we quoted Woebot saying: “I’m here to help and we can get through this together,” even though Woebot is not “together” with us in our mental health struggle any more than we and our umbrella are “together” in the rain. Mimicry seems to be the default strategy in modern chatbot development.

In this context, the focus is often shifted from the thicker concept of the therapeutic relationship to a somewhat more technical notion of *therapeutic alliance*. According to a classical characterization offered by Edward Bordin (101), therapeutic alliance consists of (a) an agreement on therapeutic goals, (b) an assignment of therapeutic tasks, and (c) the development of bonds.^4^ While it might be impossible to achieve a genuine therapeutic relationship (based on warmth, empathy, and acceptance) with a CAI, at least certain aspects of the therapeutic alliance might be reconstructed in an interaction with a chatbot. Thus, following Bordin (101), Kaveladze and Schueller (102) define the digital therapeutic alliance (DTA) as “*a user-perceived alliance* (composed of a bond, agreement on the tasks directed toward improvement, and agreement on therapeutic goals)” [*emphasis added*, pp. 88–89], which, at least to some extent, can occur in interaction with CAI. To date, at least two psychometric tools have been proposed to conceptualize and measure this aspect of interacting with chatbots (103): Mobile Agnew Relationship Measure (mARM) (104) and Digital Working Alliance Inventory (D-WAI) (105, 106). In particular, the latter – D-WAI – includes items related to therapeutic goals (e.g., “I trust the app to guide me towards my personal goals”), tasks (e.g., “I believe the app tasks will help me to address my problem”), and bonds (e.g., “The app supports me to overcome challenges”), as a way of measuring the strength of therapeutic alliance in the digital context.

In general, there is now an increasing body of research on the digital therapeutic alliance, and its overall influence on the efficacy of help provided by mental health chatbots (51, 52, 54, 56, 102–112). These studies, including randomized controlled trials, reveal varying strengths of the effects: from small up to comparable or even outperforming these found in therapy delivered by humans (as measured on traditional scales designed for assessing the strength of working alliance formed between a client and a human therapist). Thus, we can conclude that existing chatbots allow users to establish DTA, including bonding and agreeing on tasks and goals, which may contribute to reducing symptoms. Considering rapid advances in mental health chatbot development driven by studies in the field of human-computer interaction, we can expect that levels of DTA obtained in questionaries such as D-WAI or mARM will increase. Most important future improvements will likely include personalization, better adaptation of chatbots to the user’s personality, and better simulation of human characteristics (57, 108, 113–115).

Nevertheless, it is worth keeping in mind the limitations of such an approach. While psychometric tools such as mARM and D-WAI allow us to compare levels of alliance one achieves with human therapists with those one achieves with a mental health chatbot designed within the same conceptual framework, the current understanding of the DTA phenomenon is still restricted and requires further research with novel measurements. According to Lederman and D’Alfonso:

… given that such measures are more or less based on existing measures of the traditional therapeutic alliance and simply replace “therapist” with “app,” with possibly a few other minor modifications, ultimately such an approach seems unsatisfactory or incomplete, as it does not account for the possibility of certain nuances, particularities, and complexities that could arise in the context of digital interventions. Furthermore, … one would expect that not all aspects of a traditional therapeutic alliance will necessarily apply to a DTA, and that there may also be dimensions of alliance in the digital context that are not accounted for in traditional therapeutic alliance models (103) [p. 2].

Another problem worth examining is that, while mimicry might increase users’ engagement at the beginning, it might also have a detrimental effect when things do not go well, and the users’ situation is not improving despite their efforts. In such a case feeling *as if somebody cares*, might not be enough. Moreover, successfully mimicking a therapeutic relationship might be very difficult, given that it involves multiple aspects and degrees “from a sense of being provided for (the therapist will take care of me), to a safe haven (the therapist will protect me), to a solid base (life is predictable here), to a sense of coherence (the therapist understands me), to being attuned to (the therapist and I are one)” (71) [p. 49]. On the other hand, already the famous ELIZA, a simple computer program designed to mimic an interaction with a Rogerian psychotherapist, was supposedly good enough to successfully trick at least some of its users into thinking that they talk to a human therapist (116). Last but not least, mimicry raises ethical problems (28–30). In short, mimicry is a form of deception, and it is more effective the more its receivers are deceived.

The last strategy – *Emulation* – is the most demanding, and we should probably not expect it to be fully implemented anytime soon. It involves two steps. Firstly, we would have to investigate whether human–human therapeutic relationships can be analyzed as consisting of several simpler components or active ingredients, e.g., empathy, trust, positive regard, goal cohesion, understanding, etc. (95, 117–119). Secondly, we would have to try and reconstruct these components (or their counterparts) in the human-machine interaction. At this point, it is very difficult to assess to what extent this strategy is feasible. Let us take empathy as an example. While some authors claim that it is possible to build machines or chatbots capable of empathizing with people (120–123), others (124) point out that such optimism results from using an excessively restrictive characterization of empathy, e.g., identifying empathy with “empathic behavior.” But if, while developing artificial agents, we focus only on the behavioral aspect of empathy, neglecting all other aspects, such as emotional and phenomenal, maybe we are just developing more sophisticated forms of mimicry, and thus strategy three collapses to strategy two.

Finally, it might turn out to be the case that the most important active ingredient of the therapeutic relationship is something much easier to emulate or deliver by a CAI, e.g., autonomy support (125–127). According to Zuroff et al. (127), clients are “autonomously motivated when they experience themselves as having freely chosen their goals and the choice is felt to emanate from themselves” [p. 137]. Zuroff and colleagues suggest that such understood autonomy is the common factor of most efficient therapeutic interventions, and the main task of a therapist is to support rather than undermine it (128). If this is the case, CAIs may be well positioned to emulate it, simply by creating a context in which clients work through their problems without the impression of being dependent on, paternalized by, or forced into something they did not choose by an over-imposing therapist. We are yet to see whether, and in what ways, the technological solutions of the next decades will enable us to genuinely pursue the emulation strategy of solving The Problem of a Non-human Therapist.

## 5. The problem of a narrowly intelligent therapist

Based on the analysis of multiple available definitions, Shane Legg and Marcus Hutter (129) propose the following working definition of intelligence for AI research: “Intelligence measures an agent’s ability to achieve goals in a wide range of environments” [p. 402]. The kind of artificial intelligence available now can achieve goals only in a relatively constrained or “narrow” range of environments (or solve only a constrained and “narrow” range of tasks), therefore it is typically called *artificial narrow intelligence* (ANI). In recent years, the use of ANI flooded different domains of our everyday lives. It is responsible for the accuracy of our Google searches, it crushes us at chess, it transforms speech to text, it drives autonomous vehicles, and so on.

Available ANIs are becoming able to deal with more and more complex environments. Some of the most important milestones in the recent ANI development were IBM’s computer *Deep Blue* defeating the world champion in chess, and *AlphaGo* defeating (19 years later) the world champion in Go. Go is significantly more difficult for a computer to master than chess due to a gargantuan number of next possible move in any given position (130). *AlphaGo* achieved this goal, thanks to the use of the so-called *deep reinforcement learning* which combines reinforcement learning (a method of trial-and-error learning guided by reward maximization) with the use of deep neural networks (artificial neural networks using one or more hidden layers, and thus much more accurate than so-called *shallow* neural networks) (131). Even though narrow artificial intelligence is gradually becoming “less narrow,” we are still waiting for the so called artificial general intelligence (AGI), i.e., an artificial system capable of applying its intelligence to a virtually unrestricted range of tasks and environments, including ones that are new to it. This flexibility of intelligent thinking is the hallmark of human intelligence; therefore, AGI is also often referred to as *human-like AI*. While some argue that the path to AGI is relatively straightforward and we should expect to achieve this milestone within the next couple of years or decades (132), others are pessimistic about our prospects of ever building generally intelligent artificial systems (130) (for the results of an expert survey regarding this issue see (133)).^5^ Be it as it may, at this point, we are stuck with ANI, and thus the last problem on the way towards AI-based psychotherapy is the following:

**The Problem of a Narrowly Intelligent Therapist**: Can a narrowly intelligent agent conduct psychotherapy?

Imagine a complicated social game in which you are supposed to coordinate your actions with your partner in such a way that you achieve a common goal that you have previously established. To win this game, you must excel in a number of supplementary tasks (or mini games). For example, you must accurately comprehend what your partner is saying; recognize cognitive errors they are making and suggest course correction; adequately read and react to their emotions, etc. The list of mini games contributing to the success in the big game is long but closed. There will be new scenarios but no new games along the way. Finally, each of the mini games has a well-defined set of rules, and at each point you will know whether you are doing well or failing.

Solving The Problem of a Narrowly Intelligent Therapist would require assessing whether psychotherapy can be construed as such a complicated social game. Is there a long but closed list of specific tasks, mastery of which enables one to conduct psychotherapy? Can we expect narrow AI to achieve mastery of each of these tasks? We are yet to see what the answers are to these questions. One benefit of posing this problem is that it sheds light on an issue which we typically neglect in the context of training human therapists, namely, that they possess many (if not most) skills necessary to conduct psychotherapy just by virtue of successfully participating in everyday social interactions.

The second aspect of the problem discussed in this section cuts even deeper into the roots of theoretical reflection about psychotherapy. One way of thinking about talk-therapy is to think about it as a series of conversations spanning across multiple meetings. Now, here is a question: “what are these conversations about?” An answer that comes to mind is that the conversations are about whatever the client and the therapist find relevant to the client’s suffering and whatever is worth touching upon to alleviate this suffering. This might remind us of the famous first sentence of Leo Tolstoy’s novel *Anna Karenina*: “Happy families are all alike; every unhappy family is unhappy in its own way.” Just as unhappiness of unhappy families, human mental suffering comes in a myriad of different forms. Does this mean that an agent capable of conducting fully fledged psychotherapy would have to be able to engage in a meaningful conversation about all of them? If yes, we probably should not expect an ANI to be able to do it.^6^

More specifically, from the technical point of view, the task of designing a dialogue system based on ANI forces one to confront the following dilemma:

**The dialogue system dilemma**: do we want our system to be general-purpose but it’s interaction with a user uncontrollable and unpredictable, or do we want the interaction to be predictable and controllable but restricted to fulfilling a specific, narrowly defined task?

Dialogue systems based on Large Language Models, such as ChatGPT, are general-purpose. We can equally well ask them to write a carrot cake recipe, describe the history of aviation, or enumerate the species of venomous snakes living in North America.^7^ At the same time, designers of such systems have no control over the structure of the interaction – the way it unfolds depends solely on users’ prompts. On the other hand, chatbots used in fields like commerce are designed for a specific purpose. A virtual assistant on an airline website will not be able to answer questions about baking recipes or history trivia, but it will guide its user through the process of achieving a specific goal, e.g., buying a plane ticket. Many such chatbots use a predefined structure or “frame” to guide the interaction (thus their name: “frame-based” (137)). A frame-based dialogue system used on an airline’s website may fulfill the objective of acquiring all information necessary to make a reservation by filling up a virtual form with slots for TIME OF FLIGHT, DESTINATION, NUMBER OF PASSENGERS, etc. Even though they allow some flexibility (e.g., if a user starts with “I’d like to fly on Wednesday,” the chatbot might reply: “Ok, where would you like to fly on Wednesday” and if a user starts with “I’d like to fly to Toronto” the chatbot may reply with: “Ok, when would you like to fly to Toronto?”), the goal of the interaction is fixed, predefined, and narrow.

Contemporary mental health chatbots are precisely such simple frame-based systems guiding users through predesigned steps of built-in therapeutic interventions or exercises (27, 34, 138). Whenever there is a place for unscripted, natural language input from the user, a chatbot has to classify it as falling into one of the coarse-grained, pre-defined categories.^8^ It severely limits the chatbot’s ability to appropriately react to the user’s input, as witnessed, for example, by the following exchange:

1. Woebot: Okay, want to tell me more about what’s going on?
2. User: I’m feeling weird. It’s like I’m empty inside. When I look at the faces of my friends and family, I feel like they are empty as well. That’s why we cannot connect with each other. There’s nothing to hold on to.
3. Woebot: Sounds like you are dealing with feeling low…

In the future, we should expect chatbots to be able to recognize a much broader range of topics and intents brought up by users. Most likely, they will also be equipped with functionalities allowing them to learn how to respond more appropriately by learning from a user’s earlier inputs (27) and maybe even adapt to the user’s personality and thus increase their engagement (114). However, as long as we would like our ANIs to primarily implement specific therapeutic techniques, we would also have to keep the range of the topics and tasks they can engage in strictly restricted. Therefore, The Problem of the Narrowly Intelligent Therapist remains open. In light of it, we may be forced to admit either that artificial therapists are impossible to construe until we reach the level of technological engagement equal to AGI or that “psychotherapy” in the future will mean something different than it means today.

## 6. Conclusion

The use of AI is becoming increasingly widespread in the field of mental health care. In particular, the first promising attempts are being made to design AI-based technologies capable of providing psychotherapeutic help. While the available research demonstrates that mental health chatbots can, undoubtedly, be very helpful to at least some of their users, the goal of this paper was to outline the scope of the challenge of developing fully fledged AI-based psychotherapy. We offered this outline in the form of three major problems which have to be faced before we will be able to schedule our first appointments with artificial therapists. We find it very likely that in the future, each of these challenges will be overcome in one way or another. Until then, however, it is crucial to be honest and explicit about the limited role an AI can play in psychotherapeutic processes.

## Data availability statement

The original contributions presented in the study are included in the article/supplementary material, further inquiries can be directed to the corresponding authors.

## Author contributions

JPG and MH reviewed the literature, critically discussed the theoretical stance, commented on the manuscript, and approved the submitted version of the manuscript. JPG wrote the draft of the manuscript. All authors contributed to the article and approved the submitted version.

## Funding

JPG research and open access publication were supported by a grant from the Priority Research Area ‘Society of the Future’ under the Strategic Programme ‘Excellence Initiative’ at Jagiellonian University. MH research was supported by the National Science Centre, Poland (grant number: 2021/43/B/HS1/02868).

## Conflict of interest

The authors declare that the research was conducted in the absence of any commercial or financial relationships that could be construed as a potential conflict of interest.

## Publisher’s note

All claims expressed in this article are solely those of the authors and do not necessarily represent those of their affiliated organizations, or those of the publisher, the editors and the reviewers. Any product that may be evaluated in this article, or claim that may be made by its manufacturer, is not guaranteed or endorsed by the publisher.

## Footnotes

^1^Another relatively universal definition of psychotherapy comes from Norcross (33): “Psychotherapy is the informed and intentional application of clinical methods and interpersonal stances derived from established psychological principles for the purpose of assisting people to modify their behaviors, cognitions, emotions, and/or other personal characteristics in directions that the participants deem desirable” [p. 218].

^2^Based on Version 4.8.1.(214) of Woebot app. Conversational turns are numbered for reference in square brackets (so that they were not to be confused with literature references provided in parentheses).

^3^We are grateful to a reviewer for drawing our attention to MYLO and MOL.

^4^See also (30), where the alliance is elucidated as “a process where the patient and the therapist work together to determine the goals of treatment based on the patient’s existing problems and expectations from psychotherapy. Thinking together, they identify the steps to achieve that goal, forming a connection in the process” (p. 156).

^5^Notably, even the recent progress of AI technologies triggered by the development of LLMs does not guarantee a rapid arrival of AGI. As stated on the website of OpenAI — the developer of GPT-4 — “AGI could happen soon or far in the future; the takeoff speed from the initial AGI to more powerful successor systems could be slow or fast” (134).

^6^A related question that we cannot discuss here in detail is whether interaction with an ANI can be properly characterized as a “conversation” (see (135)).

^7^Nevertheless, chatbots based on LLMs are currently still helpless when confronted with sophisticated metaphors and absurdities (136) or even certain trivial logical puzzles (35).

^8^This process is called Named Entity Recognition. The “entities” it recognizes are “specific information that is extracted from the user’s input that maps the natural language phrases with the canonical phrases to understand the intent.” (27) [p. 3759].
